# Microbial metabolite ammonia disrupts TGF-β signaling to promote colon cancer

**DOI:** 10.1016/j.jbc.2025.108559

**Published:** 2025-04-29

**Authors:** Krishanu Bhowmick, Xiaochun Yang, Taj Mohammad, Xiyan Xiang, Christine L. Molmenti, Bibhuti Mishra, Srinivasan Dasarathy, Adrian R. Krainer, Md. Imtaiyaz Hassan, Keith A. Crandall, Lopa Mishra

**Affiliations:** 1 Institute for Bioelectronic Medicine, Feinstein Institutes for Medical Research, Manhasset, New York, USA; 2 Divisions of Gastroenterology and Hepatology, Department of Medicine, Northwell Health, Manhasset, New York, USA; 3 Cancer Center, Cold Spring Harbor Laboratory, Cold Spring Harbor, New York, USA; 4 Centre for Interdisciplinary Research in Basic Sciences, Jamia Nagar, New Delhi, India; 5 Department of Occupational Medicine, Epidemiology and Prevention, Zucker School of Medicine at Hofstra/Northwell, Hempstead, New York, USA; 6 Feinstein Institutes for Medical Research, Institute of Cancer Research, Manhasset, New York, USA; 7 Department of Surgery, Northwell Health, Manhasset, New York, USA; 8 Donald and Barbara Zucker School of Medicine at Hofstra/Northwell Health, Department of Neurology, Hempstead, New York, USA; 9 Division of Gastroenterology and Hepatology, Cleveland Clinic, Cleveland, Ohio, USA; 10 Computational Biology Institute and Department of Biostatistics and Bioinformatics, Milken Institute School of Public Health, George Washington University, Washington DC, USA; 11 Department of Surgery, George Washington University, Washington DC, USA

**Keywords:** Colorectal cancer, TGF-β signaling, microbiome, ammonia, βII-spectrin, SMAD3, CEACAM1

## Abstract

Colorectal cancer (CRC) is rising alarmingly in younger populations, potentially arising from factors, such as obesity, proinflammatory gut microbiome, and the accumulation of toxic metabolites. However, how metabolites such as ammonia impact key signaling pathways to promote CRC remains unclear. Our study investigates a critical link between gut microbiome alterations, ammonia, and their toxic effects on the transforming growth factor beta (TGF-**β**) signaling pathway, driving CRC progression. We observed altered microbial populations in an obesity-induced mouse model of cancer, where ammonia promotes caspase-3-mediated cleavage of the SMAD3 adaptor **β**II-spectrin (SPTBN1). Cleaved SPTBN1 fragments form adducts with ammonia that induce proinflammatory cytokine expression and disrupt TGF-**β** signaling. Extending from AlphaFold docking simulations, we identified that ammonia interacts with N-terminal SPTBN1 potentially through residues D81, Y556, S663, Y666, N986, and D1177 to form hydrogen bonds that disrupt downstream SMAD3 signaling, altering TGF-**β** signaling to a protumorigenic phenotype. Blocking SPTBN1, through an SPTBN1-specific siRNA, blocks ammonia toxicity and restores normal SMAD3/TGF-**β** signaling by reducing the abundance of SPTBN1-cleaved fragments in SW480 and Caco-2 (CRC) cell lines. In addition, our research establishes crosstalk between TGF-**β** signaling and a microbial sensor, carcinoembryonic antigen–related cell adhesion molecule 1 (CEACAM1), which is significantly overexpressed in CRC patients. We identified CEACAM1–SPTBN1 interactions at specific residues (E517 and Y520) within the immunoreceptor tyrosine–based inhibitory motif of CEACAM1 cytoplasmic domain, identifying a potential axis that is harnessed by the altered microbiome. Our study identifies mechanistic insights into how microbial metabolites target TGF-**β** as a major signaling pathway to promote CRC.

Obesity, characterized by systemic low-level inflammation, accounts for 15% to 20% of all cancers and is linked to a rising and alarming incidence of colon adenocarcinoma (colorectal cancer [CRC]) in younger adults, younger than 55 years of age (1, 2). Microbiome alterations that arise with obesity represent additional risk factors for young-onset CRC. Despite progress in early detection, with improvements in chemotherapy and immunotherapy for advanced CRC (3, 4), disparities and the changing landscape of disease are some of the challenges in this younger group (2). Gut microbial populations and their metabolites are dynamic and can shift from primarily beneficial to complex proinflammatory components and metabolites (5). For instance, metabolites such as short-chain fatty acids can induce T regulatory cell differentiation and increase the production of Th1 and Th17 cells, improving immunotherapy response (6). In contrast, another group of metabolites, secondary bile acids such as deoxycholic acid and lithocholic acid, promote cancer development (7, 8). *Fusobacterium nucleatum*–derived succinic acid inhibits the cGAS–interferon-β pathway promoting CRC through effects on the tumor microenvironment (9, 10, 11). Micronutrients, such as high vitamin D levels in combination with *Bacteroides fragilis*, enhance cancer immunity in both mice and humans that may improve antitumor immunity (12). More recent interventions targeting microbiota, such as fecal microbiota transplantation and antibiotics, are also promising (13, 14). One example is the community-wide eradication of *Helicobacter pylori* using a 10-day antibiotic regimen (omeprazole, tetracycline, metronidazole, and bismuth citrate) found to reduce gastric cancer risk (14). However, these approaches may result in resistance to antimicrobial therapies as well as significant toxicities. Therefore, targeting strategies to specific microbial metabolites through delineating their interactions with cancer signaling pathways may provide a more attractive approach.

A specific accumulation of ammonia derived from the microbiota, together with decreased cell autonomous metabolism, has recently been demonstrated to promote CRC through a predominantly T-cell–dependent mechanism (15). High ammonia levels induce T-cell oxidative stress through dysregulation of the transsulfuration pathway (15). Subsequent T-cell exhaustion, a reduction in T-cell proliferation, and a decrease in the ability to clear the toxic waste products promote CRC (15). In addition, ammonia impairs tight junction barriers by inducing mitochondrial dysfunction in CRC cells (16). Yet, how ammonia and other metabolites target specific signaling pathways, altering them to promote CRC is only partially understood.

We have observed altered microbiomes in our mouse models with disruption of transforming growth factor beta (TGF-β) signaling that develop spontaneous CRC and other gastrointestinal cancers (17, 18, 19). Interestingly, our group and others have observed that these mutant mice do not develop cancers in a germ-free environment (17, 18, 20, 21, 22). Examples include mouse models of CRC with haploinsufficiency of *Tgfbr2*, *Smad4*, and intercrosses between *Smad3/4* with the adaptor *Sptbn1 (Tgfbr2*^−/−^, *Smad4*^+/−^*Sptbn1*^+/−^, and *Smad3*^+/−^*Sptbn1*^+/−^ on a C57BL/6 background) (17, 18, 22, 23). More recently, we have uncovered obesity-driven cancers in our mouse models with disruption of TGF-β signaling and loss of aldehyde dehydrogenase 2 (Aldh2) (24, 25). ALDH2 detoxifies cells of lipid end products—reactive aldehydes such as 4-HNE that accumulate with a high-fat diet (HFD), and the *Aldh2*^*−/−*^*Sptbn1*^*+/−*^ mice provided new insight into the role of obesity in promoting cancer (25). However, how the altered microbial populations in these mutant mice with disruption of TGF-β signaling promote cancer remains unclear.

For potential sensors that the altered microbial populations could signal to in a TGF-β-dependent manner, we sought to further examine the carcinoembryonic antigen–related cell adhesion molecule (CEACAM) family of proteins (17, 26, 27). CEACAM1, like TGF-β, plays dual role in immune modulation and cancer, has been shown to interact with TGF-β members, is important for activation of CD8+ T cells, and the absence of CEACAM1 on virus-specific CD8+ T cells limits the antiviral CD8+ T-cell response (28). CEACAMs have been identified as receptors/sensors for bacterial docking and entry. For instance, CEACAM1 binds to *F. nucleatum via* the trimeric autotransporter adhesin CbpF, inhibit T-cell response, support immune evasion of cancer cells, and promote tumorigenesis (29). High CEACAM1 abundance in CRC, liver (hepatocellular carcinoma), melanoma, gastric, thyroid, and bladder cancer is associated with invasion and an increase in metastasis (30, 31). We therefore hypothesized that crosstalk between altered microbiomes, accumulated toxic metabolites, such as ammonia, disrupts normal CEACAM1–TGF-β signaling in gut epithelial cells promoting oncogenesis observed in CRC. For this, we explored the microbiomes of HFD-fed *Aldh2*^*−/−*^*Sptbn1*^*+/−*^ mice, which we refer to as obese TGF-β-driven mouse model of cancer (OTM). Next, we examined how ammonia exerts toxic effects on the TGF-β pathway, and the mechanisms by which luminal bacteria could transmit signaling through the CEACAM–TGF-β axis to promote CRC.

## Results

### Alterations in the TGF-**β** pathway have an association with poor survival of CRC patients; diet induces proinflammatory gut microbiota in an OTM

**Figure 1:**
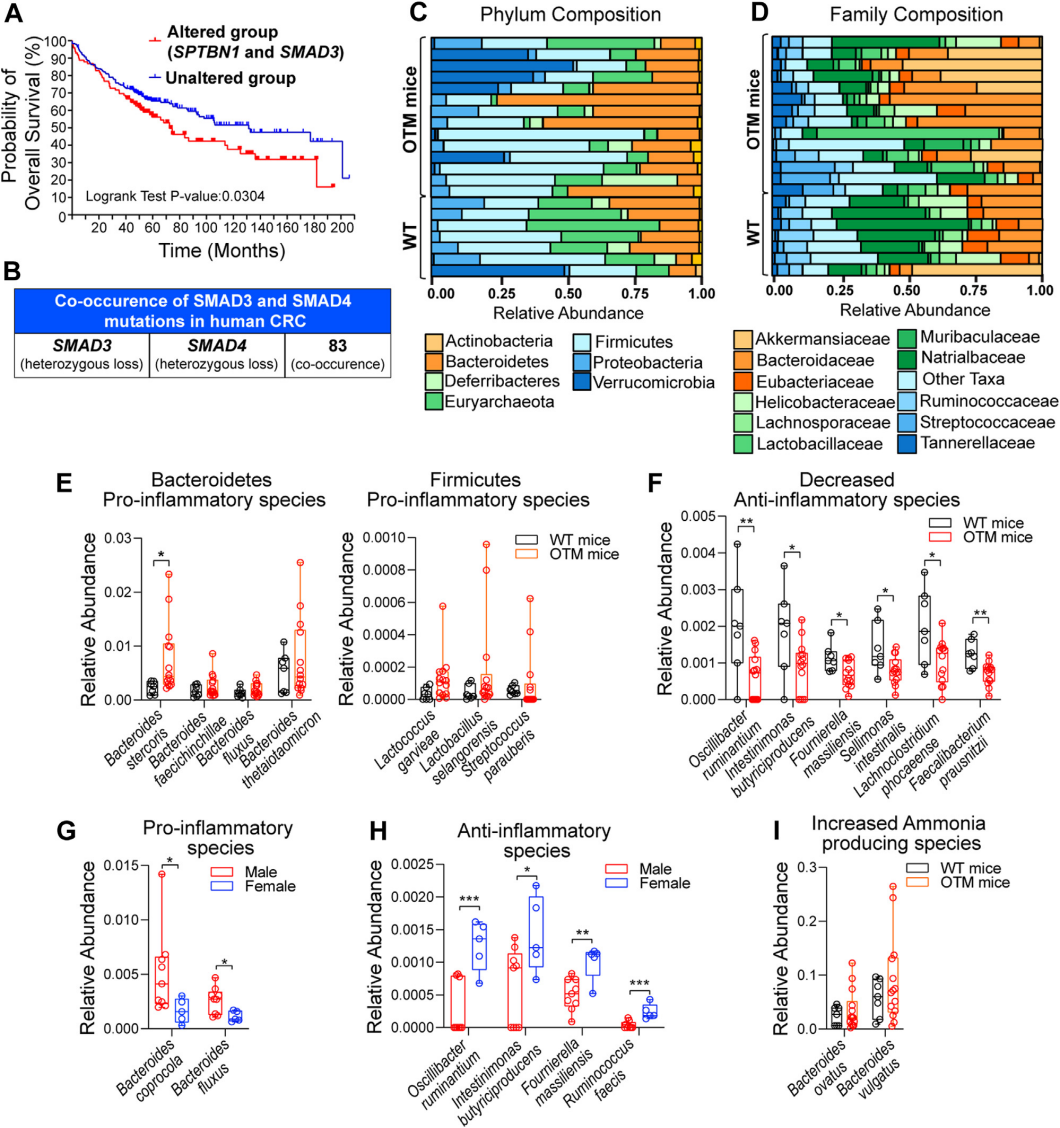
**SPTBN1 and SMAD3 alterations are associated with poor survival of CRC patients.** Alterations in gut microbiome in obese TGF-β-driven mice (OTM) compared with WT mice. *A*, Kaplan–Meier curves illustrating the overall survival in *SPTBN1*^MUT^-*SMAD3*^MUT^ group (altered group) and unaltered group in CRC cohorts. *p* Value is calculated by log-rank test. *B*, frequent co-occurrences of *SMAD3* and *SMAD4* mutations are observed in CRC patients. *C* and *D*, shotgun metagenomic analysis of fecal samples collected from WT and OTM mice, reveal the relative abundance of microbial communities at the phylum and family levels. *E*, OTM mice exhibited an increased proinflammatory microbiome species from the Bacteroidetes and Firmicutes phyla compared with WT mice. *F*, conversely, anti-inflammatory microbiome species were significantly reduced in OTM mice (n = 14) compared with WT mice (n = 7). *G* and *H*, sex-specific altered microbiome profiles in obese TGF-β driven mouse model (OTM). Proinflammatory microbial species are more abundant in male than female OTM mice, whereas anti-inflammatory species are higher in female OTM mice. *I*, a marked increase in ammonia-producing bacteria was also observed in OTM mice relative to WT mice. Data are presented as the relative abundance (*y*-axis) of bacterial species (*x*-axis). Statistical significance was determined using an unpaired *t* test, with ∗*p* < 0.05 indicating significance. CRC, colorectal cancer; SPTBN1, SMAD3 adaptor βII-spectrin; TGF-β, transforming growth factor beta.

We previously reported that nearly 65% of colon adenocarcinoma cases from the pan cancer cohort in The Cancer Genome Atlas (TCGA) exhibit alterations in TGF-β pathway genes (32), whereas mice with defects in TGF-β signaling (*Sptbn1*^+/−^*Smad3*^+/−^ and *Smad4*^+/−^*Sptbn1*^+/−^) spontaneously developed adenomas and CRC (17, 33). Here, we first evaluated the impact of TGF-β pathway alterations on patient outcomes, utilizing a separate cohort of 348 CRC patients (Sidra-LUMC AC-ICAM) (34). Kaplan–Meier (K–M) survival analysis demonstrated a significant reduction in overall survival among 28% patients with a heterozygous loss of *SPTBN1* and/or *SMAD3* (Fig. 1*A*; *p* < 0.05). In addition, a statistically significant co-occurrence of *SMAD3* and *SMAD4* heterozygous loss also occurred within this CRC cohort (Fig. 1*B*; *p* < 0.001). These findings suggest that heterozygous loss of *SPTBN1*, *SMAD3*, and *SMAD4* may synergistically promote colorectal carcinogenesis and contribute to poor prognoses in CRC patients.

Analysis of the fecal microbiota in OTM mice with exposure to an HFD reveals a notable shift toward proinflammatory species within the Bacteroidetes and Firmicutes phyla, in comparison to WT controls (Fig. 1*C*). At the family level, Bacteroidaceae and Akkermansiaceae are dominant in OTM mice (Fig. 1*D*). Specific bacterial species, such as *Bacteroides stercoris*, *Bacteroides fluxus*, *Bacteroides thetaiotaomicron*, *Lactococcus garvieae*, *Lactococcus selangorensis*, and *Streptococcus parauberis*, are highly enriched in OTM mice in comparison to WT mice on an HFD (Fig. 1*E*). Interestingly, *B. stercoris*, a species with a connection to human CRC (35), is significantly increased in OTM mice. In contrast, commensal anti-inflammatory gut microbes associated with a healthy microbiome, such as *O. ruminantium*, *F. massiliensis*, and *F. prausnitzii*, show a marked decrease in OTM mice (Fig. 1*F*). Notably, sex-biased microbiome alterations show that proinflammatory species, such as *Bacteroides coprocola* and *B. fluxus*, were more prevalent in male OTM mice, whereas female OTM mice had higher levels of anti-inflammatory species (Fig. 1, *G* and *H*). Furthermore, ammonia-producing microbial species, including *Bacteroides ovatus* and *Bacteroides vulgatus*, increase in OTM mice, linking these microbial shifts with increased ammonia production (Fig. 1*I*). These findings underscore the impact of HFD/Western diet (WD) on the gut microbiota in TGF-β-regulated models of obesity and susceptibility to cancer, with distinct species-specific changes.

### Microbial metabolite ammonia interacts with SPTBN1, forming toxic adducts that divert TGF-**β** signaling to a pro-oncogenic role

Recent studies show that ammonia can directly bind the cholesterol-sensing protein SCAP at residue D428, inducing conformational rearrangements and reducing SCAP interactions with Insig, altering downstream signaling (36). We therefore hypothesized that ammonia may similarly interact with βII-spectrin (SPTBN1), a critical SMAD3 adaptor disrupting TGF-β signaling.

**Figure 2:**
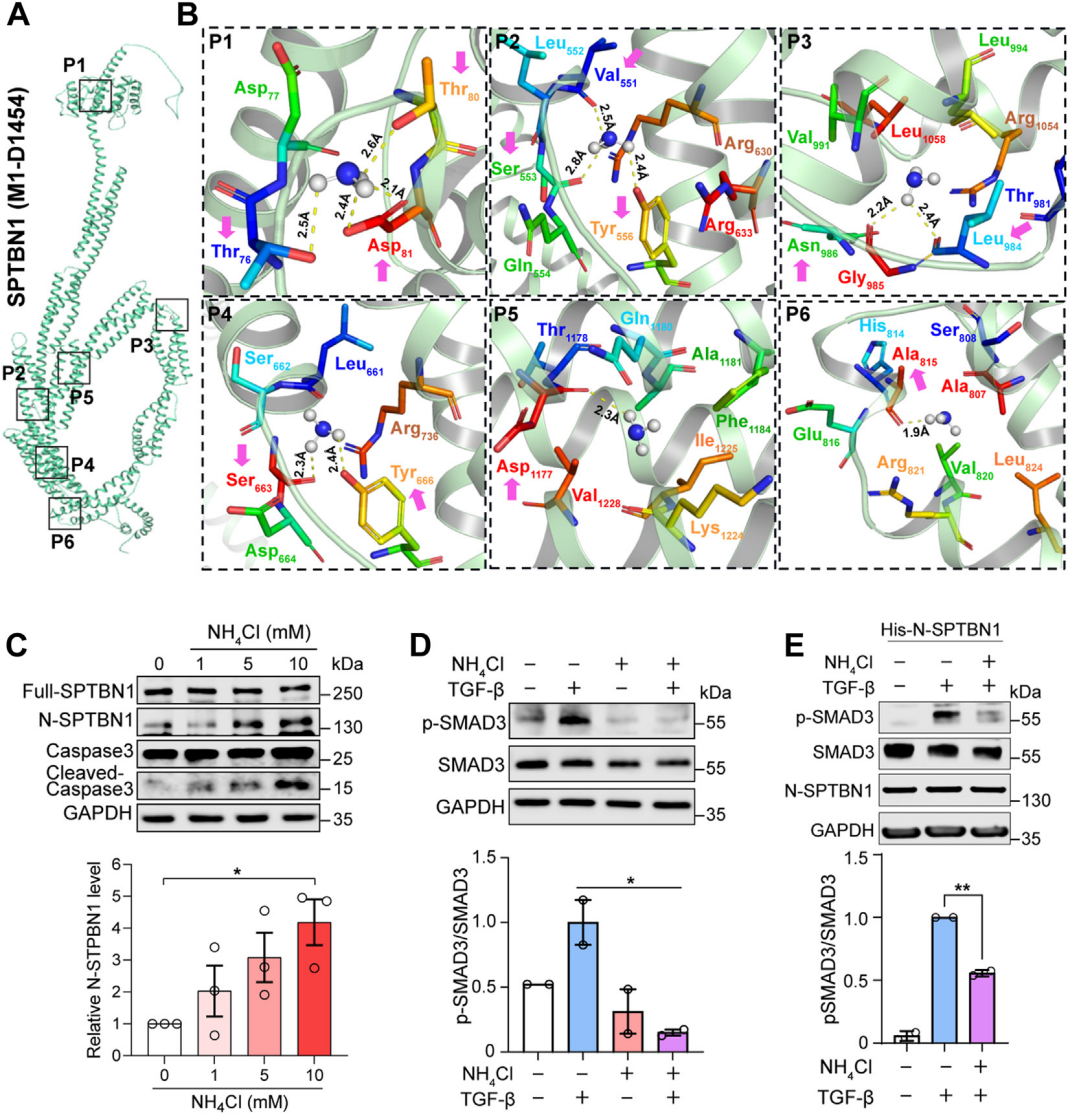
**Microbial metabolite ammonia alters TGF-β signaling to a pro-oncogenic phenotype.***A*, potential ammonia-binding sites on SPTBN1 (M1-D1454) using AlphaFold2-based molecular docking analyses. *B*, ammonia binds SPTBN1 through multiple hydrogen bonds represented in *yellow dashes*. Hydrogen bonds are formed within 3.5 Å of distance. *Right-magnified panels* show polar contacts with varying distances between ammonia and SPTBN1. These illustrations were generated through PyMOL using the preferential (P1) and alternative (P2–P6) docked poses of ammonia with SPTBN1 (M1-D1454). SPTBN1 residues involved in hydrogen bond formation with ammonia are indicated by *pink* arrows. *C*, Western blot analyses reveal dose-dependent ammonia-induced cleavage of SPTBN1 by caspase-3 in HCT116 cells. Results are the average representative of three independent experiments. *D*, ammonia reduces pSMAD3 expression in SW480 cells treated with NH_4_Cl (10 mM) and TGF-β (200 pM). *E*, overexpression of N-SPTBN1 exacerbates ammonia-induced TGF-β signaling disruption by reducing p-SMAD3 expression. Results are the average representative of two independent experiments. Data are presented as mean ± SD. Statistically significant changes relative to TGF-β-treated controls are indicated (∗*p* < 0.05). SPTBN1, SMAD3 adaptor βII-spectrin; TGF-β, transforming growth factor beta.

To examine how ammonia interacts with N-terminal SPTBN1, we first utilized AlphaFold-based structural modeling and performed molecular docking analyses. Docking results reveal strong binding affinities of ammonia with SPTBN1, with 13 polar interactions forming within 3.5 Å of key residues (T76, T80, D81, V551, S553, Y556, S663, Y666, A815, L984, N986, and D1177) that distribute across several spectrin repeats (Fig. 2, *A* and *B*). These findings suggest that ammonia may bind to SPTBN1-cleaved fragments, potentially interfering with downstream signaling functions.

To determine the mechanism of how ammonia induces toxicity with SPTBN1-cleaved fragments, we embarked upon further studies. We first observed that ammonia activates caspase-3 in a dose-dependent manner in HCT116 human colon cancer cells, leading to an increase in SPTBN1-cleaved fragments, with the highest cleavage observed at 10 mM NH_4_Cl (Fig. 2*C*). To assess downstream signaling, we treated CRC cells with ammonia and TGF-β and assessed phosphorylated SMAD3 (p-SMAD3) expression. Western blot analyses confirm significantly reduced p-SMAD3 levels under ammonia-treated cells compared with control TGF-β-only treated cells (Fig. 2*D*).

To define a clear role of SPTBN1-cleaved fragments in ammonia toxicity, we overexpressed N-SPTBN1 in human colon cancer cells (SW480), followed by treatment with ammonia (NH_4_Cl) and TGF-β (Fig. 2*E*). Quantitative RT–PCR and Western blot analyses confirm significant overexpression of N-SPTBN1 in CRC cells (Fig. S1). Overexpression of N-SPTBN1 fragment significantly reduces p-SMAD3 levels, suggesting that N-SPTBN1, in the presence of ammonia, disrupts SMAD3 activation. These results indicate that ammonia disrupts TGF-β signaling by inducing cleavage of SPTBN1 and reducing p-SMAD3 expression in human colon cancer cells.

### SPTBN1 silencing restores tumor-suppressive TGF-**β** signaling

To understand ammonia toxicity on TGF-β signaling, we examined the impact of ammonia on expression of TGF-β/SMAD3-regulated genes. Indeed, RNA-sequencing analyses revealed altered expression of TGF-β/SMAD3-regulated genes, activation of oncogenic TGF-β signaling (*TGF-β1*, *MYC*, *etc.*), in ammonia-treated cells, and these changes were more pronounced in cells with exposure to both ammonia and TGF-β in comparison to controls (Fig. S2).

Previously, we have identified that endogenous toxins, such as reactive aldehydes produced by a WD, disrupt TGF-β signaling, contributing to inflammation and cancer, and that siRNA-mediated silencing of SPTBN1 blocks these toxic effects (24, 25).

**Figure 3:**
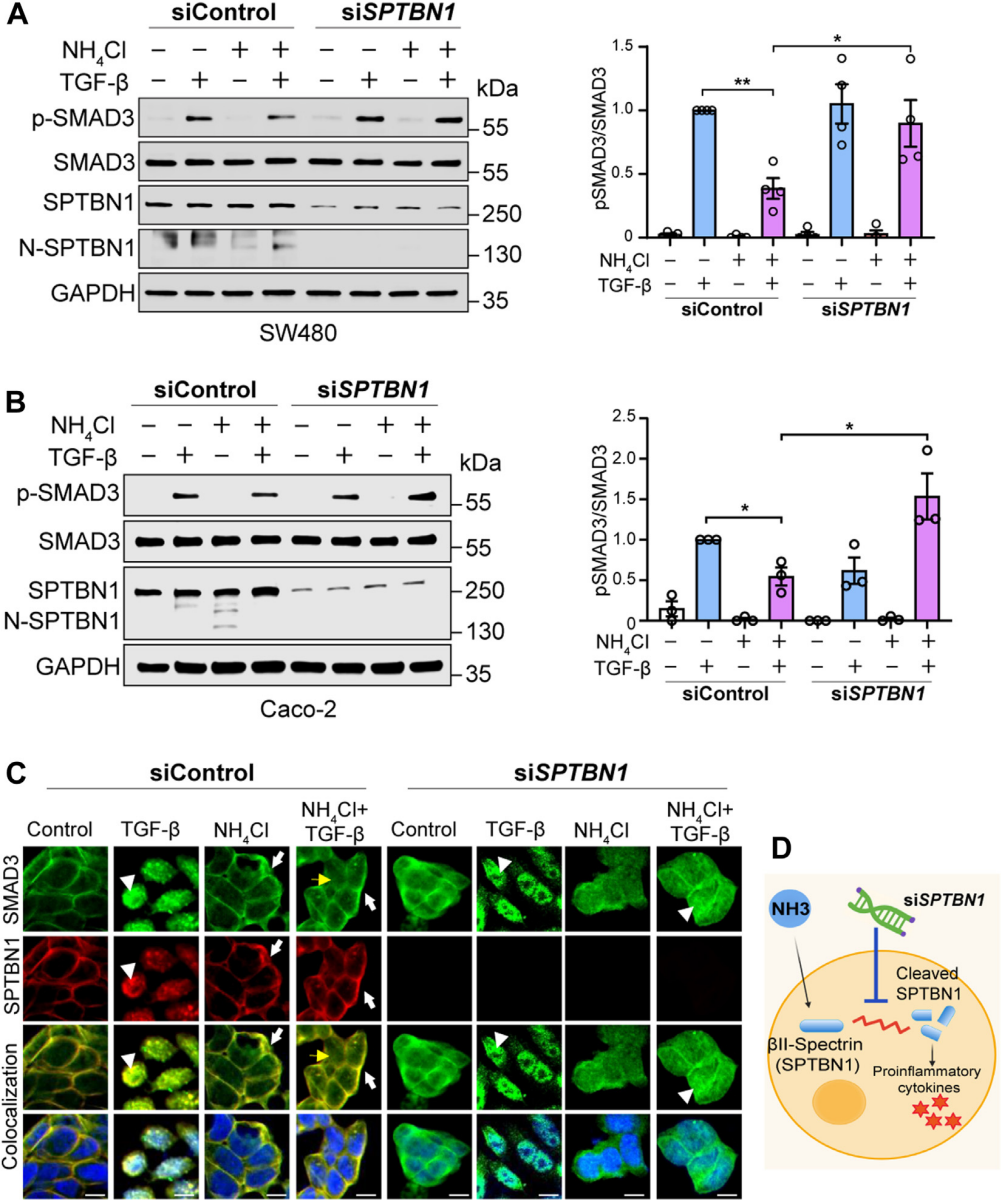
**SPTBN1 knockdown using siRNA blocks ammonia toxicity and restores TGF-β signaling.***A* and *B*, Western blot analyses of indicated proteins in SW480 cells (*A*, *top panel*) and Caco-2 cells (*B*, *lower panel*) treated with si*SPTBN1* and exposed to NH_4_Cl (10 mM) and TGF-β (200 pM). Quantification of the p-SMAD3/SMAD3 ratio from four (SW480 cells) and three (Caco-2 cells) independent experiments. Quantification of pSMAD3 protein levels through the densitometry analysis is presented on the *right*. Data are presented as mean ± SD. Statistical significance was evaluated by Student’s *t* test. ∗*p* < 0.05 and ∗∗*p* < 0.01. *C*, SPTBN1 silencing using si*SPTBN1* blocks ammonia toxicity and partially restores SMAD3 nuclear localization. *Arrowheads* indicates SMAD3 nuclear localization. *White* and *yellow arrow* indicates SMAD3 localization at the membrane and in the cytoplasm, respectively. Scale bar represents 20 μm. *D*, proposed model illustrating how si*SPTBN1* blocks ammonia toxicity and restores normal TGF-β signaling. SPTBN1, SMAD3 adaptor βII-spectrin; TGF-β, transforming growth factor beta.

Therefore, we investigated the role of SPTBN1-cleaved fragments in ammonia-mediated alterations in TGF-β–SMAD3 signaling, utilizing siRNA targeting *SPTBN1* (si*SPTBN1*) in SW480 and Caco-2 human colon cancer cells and treated cells with ammonia. We achieved 80% knockdown efficiency using si*SPTBN1* in both SW480 and Caco-2 cells (Fig. S3). Ammonia treatment alone significantly reduced p-SMAD3 levels, as detected by Western blot analyses (Figs. 3, *A* and *B*, and S4). In contrast, *SPTBN1* silencing blocked ammonia toxicity and restores SMAD3 phosphorylation, counteracting the toxic effect of ammonia on SMAD3 signaling (Fig. 3, *A* and *B*). Next, we performed RNA-sequencing analyses on SW480 human colon cancer cells treated with ammonia and TGF-β following SPTBN1 knockdown. Gene set enrichment analyses revealed that CRC-related genes are downregulated in cells receiving si*SPTBN1* treatment when treated with ammonia and TGF-β, compared with siControl-treated cells (Fig. S5).

To further investigate the impact of ammonia on TGF-β signaling, we examined the subcellular localization of SMAD3 using confocal microscopy. Under TGF-β stimulation, SMAD3 robustly translocated to the nucleus, consistent with its activation and transcriptional role (Fig. 3*C*, *second panel*). In contrast, in cells cotreated with ammonia and TGF-β, SMAD3 is predominantly expressed in the cytoplasm and at the membrane (Fig. 3*C*, *fourth panel*). SPTBN1 silencing using si*SPTBN1* blocks ammonia toxicity and partially restores TGF-β-induced normal SMAD3 nuclear localization and signaling (Fig. 3*C*, *last panel*). These findings suggest that SPTBN1 silencing can alleviate ammonia toxicity, attenuate oncogenic transcriptional programs, and restore tumor-suppressive TGF-β–SMAD3 signaling (Figs. 3*D* and S6).

### Raised CEACAM1 levels are observed in CRC that co-occur with Smad3 alterations

**Figure 4:**
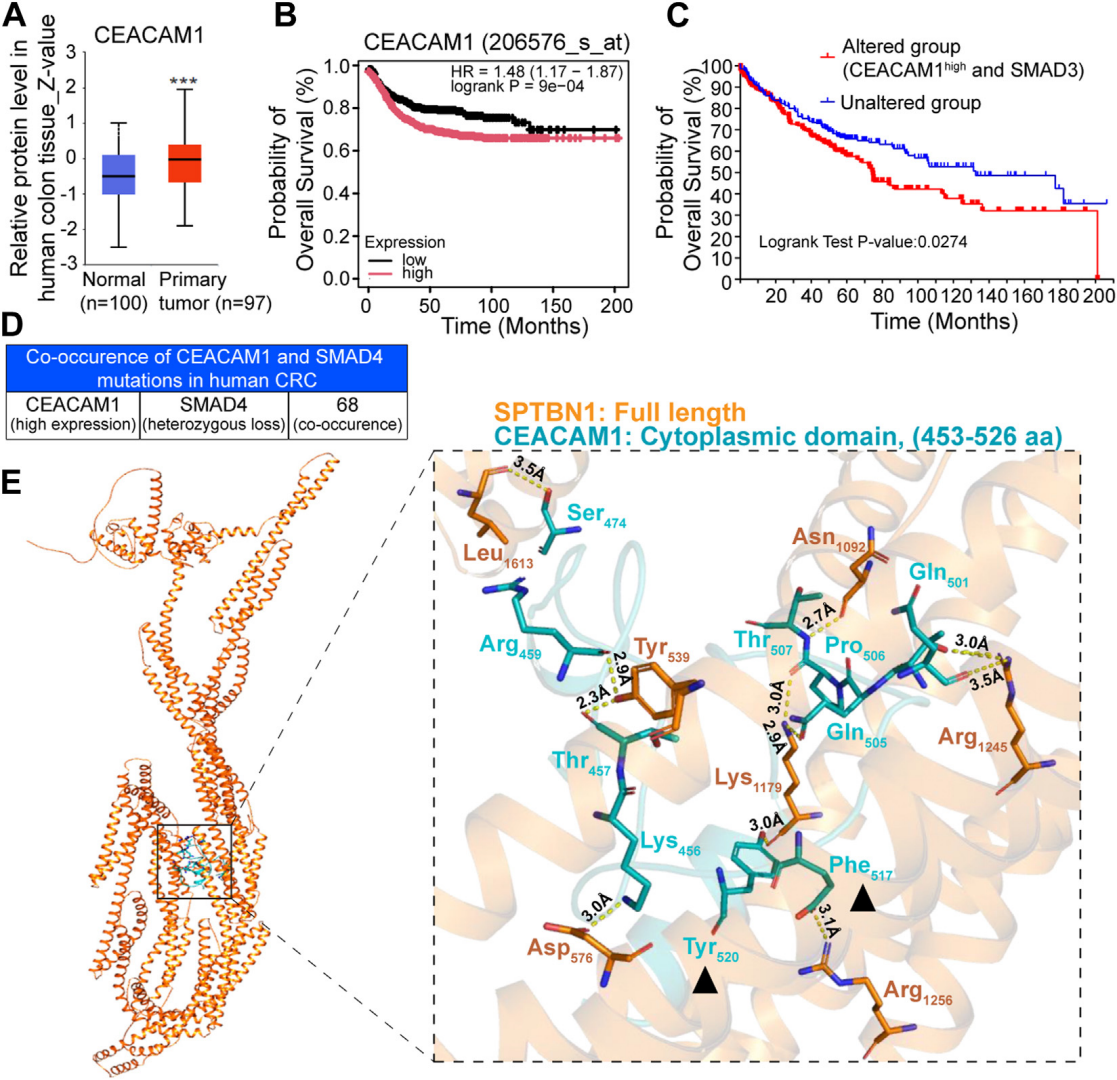
**High CEACAM1 levels and SMAD3 alterations are associated with significantly poorer overall survival in CRC patients.***A*, high CEACAM1 protein levels are observed in CRC patients. *B*, Kaplan–Meier survival analysis demonstrates that CRC patients with high CEACAM1 protein expression have significantly reduced overall survival compared with those with low CEACAM1 expression. *C*, frequent co-occurrences of increased CEACAM1 with Smad3/4 mutations are observed in CRC patients. *D*, Kaplan–Meier curves illustrating the overall survival in CEACAM1^high^–*SMAD3*^MUT^ group (altered group) and unaltered group in TCGA CRC cohorts. Overall *p* value is calculated by log-rank test. Data are presented as mean ± SD. ∗*p* < 0.05, ∗∗*p* < 0.01, and ∗∗∗*p* < 0.001. *E*, structural insights reveal specific interaction sites between SPTBN1 and the CEACAM1 cytoplasmic domain. Analyses of SPTBN1 and CEACAM1 cytoplasmic domain (amino acids 453–526) interaction through AlphaFold-based molecular docking. Enlarged region shows predicted interacting residues between SPTBN1 (*orange*) and CEACAM1 (*blue*). *Black arrowhead* indicates the ITIM residues involved in interaction with SPTBN1. CEACAM1, carcinoembryonic antigen–related cell adhesion molecule 1; CRC, colorectal cancer; ITIM, immunoreceptor tyrosine–based inhibitory motif; SPTBN1, SMAD3 adaptor βII-spectrin; TCGA, The Cancer Genome Atlas.

Our previous discovery reveals a close correlation between the expression of CEACAM family members and TGF-β pathway activity in TCGA colorectal adenocarcinoma patient cohort (17). Here, we investigated the relationship between alterations in TGF-β pathway genes, expression of CEACAM1, and patient outcome using the human colon cancer dataset from TCGA. CEACAM1 protein abundance was significantly elevated in CRC compared with normal tissues (Fig. 4*A*). Importantly, CRC patients with high CEACAM1 expression had poorer prognoses and lower survival probabilities (Fig. 4*B*). Moreover, CRC patients with high CEACAM1 expression and *SMAD3* genomic alterations (heterozygous loss and missense mutations) exhibited a significantly lower 10-year overall survival rate, compared with those in the unaltered group (37% *versus* 52%) (Fig. 4*C*). In addition, we observed a significant co-occurrence between high CEACAM1 expression and heterozygous loss of *SMAD4* gene in the CRC patients (*p* < 0.05) (Fig. 4*D*). Together, these findings highlight a potential synergistic relationship between elevated CEACAM1 expression and disrupted TGF-β signaling components, such as SMAD3 and SMAD4, in driving poor clinical outcomes in CRC.

### CEACAM1 interacts with SMAD3 adaptor SPTBN1

Human CEACAM1 is a type I transmembrane protein, containing an N-terminal immunoglobulin-like domain (IgV), a transmembrane region, and a cytoplasmic domain that transmits intracellular signaling. Molecular modeling based on AlphaFold predictions identified key residues in the cytoplasmic domain (453–526 amino acids) of CEACAM1 and SPTBN1 that are involved in their interaction (Fig. 4*E*). Stability of the SPTBN1–CEACAM1 complex was strengthened by formation of 10 hydrogen bonds, which include seven residues from N-terminal region of SPTBN1 and 11 residues within CEACAM1 cytoplasmic domain. Specifically, K456, T457, R459, E517, and Y520 in immunoreceptor tyrosine–based inhibitory motif (ITIM) of CEACAM1 are possible residues involved in interactions with SPTBN1. Furthermore, coimmunoprecipitation experiments confirmed this interaction (Fig. S7).

**Figure 5:**
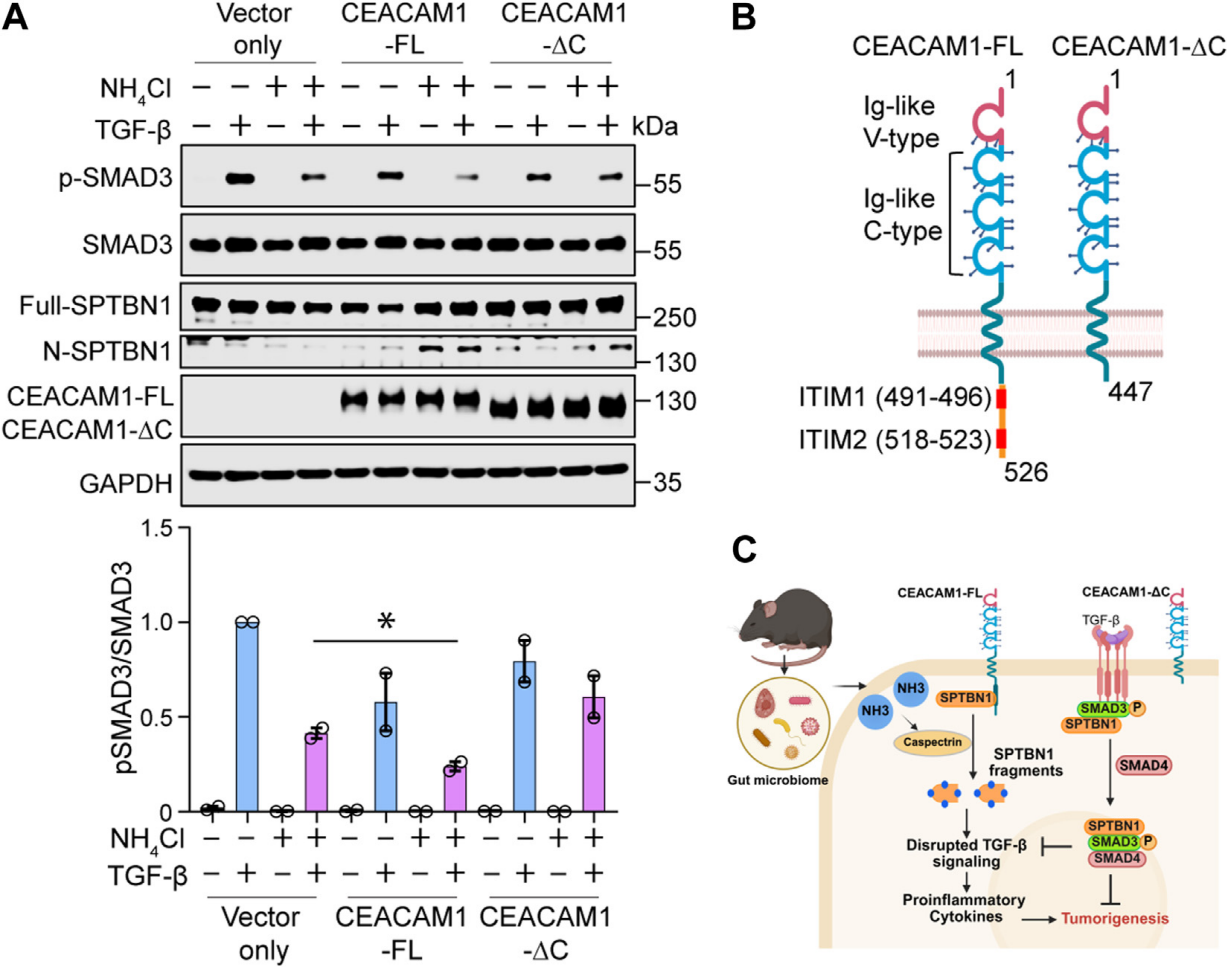
**CEACAM1 cytoplasmic domain contributes to ammonia-induced disruption of TGF-β signaling in human colon cancer cells.***A*, Western blot analyses of p-SMAD3 expression in SW480 human colon cancer cells overexpressing either full-length CEACAM1 (CEACAM1-FL) or CEACAM1 C-terminal deletion mutant (CEACAM1-ΔC). Cells were treated with NH_4_Cl (10 mM) and/or TGF-β (200 pM) as indicated. GAPDH was used as a loading control. Quantification of the p-SMAD3/SMAD3 ratio from two independent experiments. Data are presented as mean ± SD. Statistical significance was evaluated by Student’s *t* test. ∗*p* < 0.05. *B*, schematic representation of CEACAM1-FL and a C-terminal deletion mutant (CEACAM1 ΔC) with individual domain information. *C*, schematic diagram of (*A*). CEACAM1-FL, but not CEACAM1-ΔC, interacts with SPTBN1 through its cytoplasmic domain to regulate SMAD3 phosphorylation in response to ammonia exposure, thereby disrupting TGF-β signaling. CEACAM1, carcinoembryonic antigen–related cell adhesion molecule 1; TGF-β, transforming growth factor beta.

We further investigated the role of CEACAM1 in ammonia-mediated disruption of TGF-β signaling, by examining p-SMAD3 in SW480 human colon cancer cells after overexpressing either full-length CEACAM1 (CEACAM1-FL) or a C-terminal deletion mutant lacking the ITIM motif (CEACAM1-ΔC) (Fig. 5, *A* and *B*). We observed a significant reduction in p-SMAD3 expression in CEACAM1-FL–expressing SW480 cells upon ammonia treatment, compared with control cells and cells expressing the CEACAM1-ΔC terminal fragment (Fig. 5*A*). In addition, we found an increase in spectrin cleavage product (N-SPTBN1) in CEACAM1-FL–expressing cells compared with CEACAM1-ΔC–expressing cells (Fig. 5*A*). These findings suggest that CEACAM1 interacts with SPTBN1 through its cytoplasmic domain and that this interaction is important for ammonia toxicity (Fig. 5*C*).

## Discussion

The relationship among diet, the microbiome, and the immune system is increasingly considered as a significant factor in immunity and cancer (37, 38, 39). Despite our progressive understanding on gut commensal’s role in effectiveness of immune checkpoint blockade therapy (12, 38, 39), specific host factors that enable gut-residing microbes to impact these anticancer immune reactions are still not well understood. Our results demonstrate that diet-induced alterations in the gut microbial population with accumulation of toxic metabolites, such as ammonia, impair normal CEACAM1–TGF-β signaling, lead to a pathological switch, promoting tumorigenesis. In obese individuals, the gut microbiota is reflected by higher levels of Firmicutes and an overall decline in microbial genetic diversity (40). Our results reveal that HFD-fed obese TGF-β-driven mice (OTM) exhibit a distinct microbiota composition, with a significant increase in proinflammatory Bacteroides and Firmicutes. In addition, we observed a significant decrease in commensal anti-inflammatory bacteria in OTM mice. In particular, *B*. *stercoris* was enriched both in OTM mouse model and human patients with CRC, suggesting that gut microbiota alteration induced by HFD in OTM mouse model shared some similarity with characteristics of patients with CRC (35). The findings from the human TCGA dataset correlates with the genetic and microbiome alterations observed in the OTM mouse model, where there was a significant co-occurrence of alterations in *SPTBN1* and *SMAD3* or *SMAD3* and *SMAD4*, which were strongly associated with poor overall survival in CRC patients. The mouse and human data suggest that SPTBN1 and SMAD3 play critical roles not only in TGF-β signaling but also in mediating the inflammatory and tumor-promoting effects of a dysregulated gut microbiome.

Sex-specific alterations in the gut microbiome of OTM mice provide intriguing insights into the differential roles of microbial species in male and female CRC patients. Proinflammatory species, such as *B. coprocola*, were more prevalent in male mice, whereas females showed higher levels of anti-inflammatory species. These differences may be influenced by hormonal variations or sex-specific immune responses that shape microbial colonization in the gut. Estrogen, for example, shows the ability to enhance the abundance of beneficial microbial species that promote gut health, whereas testosterone is associated with increased gut permeability and a higher inflammatory response (41). Moreover, females typically exhibit stronger immune responses, which could support a microbiome rich in anti-inflammatory species, whereas males often experience more pronounced inflammatory responses, increasing the likelihood of harboring proinflammatory microbes (41). These hormonal influences could explain the higher prevalence of proinflammatory bacteria in male OTM mice, making them more susceptible to CRC development.

Previously, we observed that HFD/stress conditions lead to proinflammatory stress from high concentrations of lipid metabolites/reactive aldehydes, such as 4-HNE, activate caspase-3, leading to βII-spectrin cleavage products. Subsequently, the toxic spectrin cleaved products altered normal TGF-β signaling, stimulated lipogenesis, and promoted cancer (24, 25). This pathological caspase-mediated pathway in mice causes inflammation and susceptibility to cancer in mice on a WD. Our findings reveal a novel mechanism by which the microbial metabolite ammonia disrupts tumor-suppressive TGF-β signaling *via* structural and functional modification of SMAD3 adaptor SPTBN1. Here, we observe that microbial metabolite ammonia activates caspase-3, leading to the cleavage of SPTBN1 and the formation of toxic ammonia–cleaved SPTBN1 adducts, which divert TGF-β signaling, reducing p-SMAD3 levels, and switch normal signaling to a pro-oncogenic signaling. Pro-oncogenic properties of ammonia in CRC have been described before, but alterations in signaling pathways are unclear (15, 36). We observed that N-terminal SPTBN1 interacts with ammonia and disrupts TGF-β signaling. These studies support molecular docking analyses where we identified multiple high-affinity binding residues across the spectrin repeats of SPTBN1 (*e.g.*, T76, T80, D81, V551, S553, Y556, S663, Y666, A815, L984, N986, and D1177), which could form stable adducts with ammonia and disrupt its critical function as SMAD3 adaptor in TGF-β signaling, promoting oncogenesis. This interaction highlights the broader role of microbial dysbiosis in CRC, where shifts in the microbial populations result in the production of toxic metabolites. We reveal that reducing the abundance of SPTBN1 through siRNA inhibits ammonia–SPTBN1 adduct formation and restores tumor-suppressive TGF-β signaling.

This study demonstrates that high CEACAM1 expression co-occurs with TGF-β signaling components (SMAD3 and SMAD4) in CRC, correlating with a poorer prognosis. Human CEACAMs have been identified as receptors that Gram-negative bacteria use for docking and entry. Some of the characterized bacterial adhesins that dock and enter epithelial cells through CEACAM1 encompass CbpF from *Fusobacterium* spp., Opa from *Neisseria* spp., HopQ from *H. pylori*, and UspA1 from *Moraxella catarrhalis* (29, 42, 43, 44). OTM mice is enriched in several Gram-negative bacterial species, including *B. stercoris*, *B. fluxus*, and *B. thetaiotaomicron*. Our findings suggest that elevated CEACAM1 expression in CRC patients with disrupted TGF-β signaling facilitates increased microbial adhesion and entry, potentially exploited by altered microbiome observed in the OTM microbiota.

In this study, we discovered a functional link between CEACAM1 and SPTBN1 in the context of ammonia toxicity. CEACAM1 is the only member of the CEACAM family to possess an ITIM at the C-terminal region. Phosphorylated ITIMs recruit SHP-1 and SHP-2 phosphatases, which inactivate receptor tyrosine kinases or adaptor proteins by dephosphorylating their tyrosine residues at the cell surface (45, 46). We also find that SPTBN1 interacts with CEACAM1 cytoplasmic domain in colon cancer cells. Interestingly, E517 and Y520 in ITIM motif are possible residues involved in this interaction. Importantly, cells expressing CEACAM1-FL with an intact C-terminal domain showed a significant reduction in SMAD3 phosphorylation upon ammonia treatment, whereas this effect was lost in cells expressing a CEACAM1 mutant lacking the C-terminal ITIM domain. This indicates that the CEACAM1-FL protein, specifically its C-terminal ITIM motif, is required for mediating ammonia-induced disruption of TGF-β signaling.

**Figure 6:**
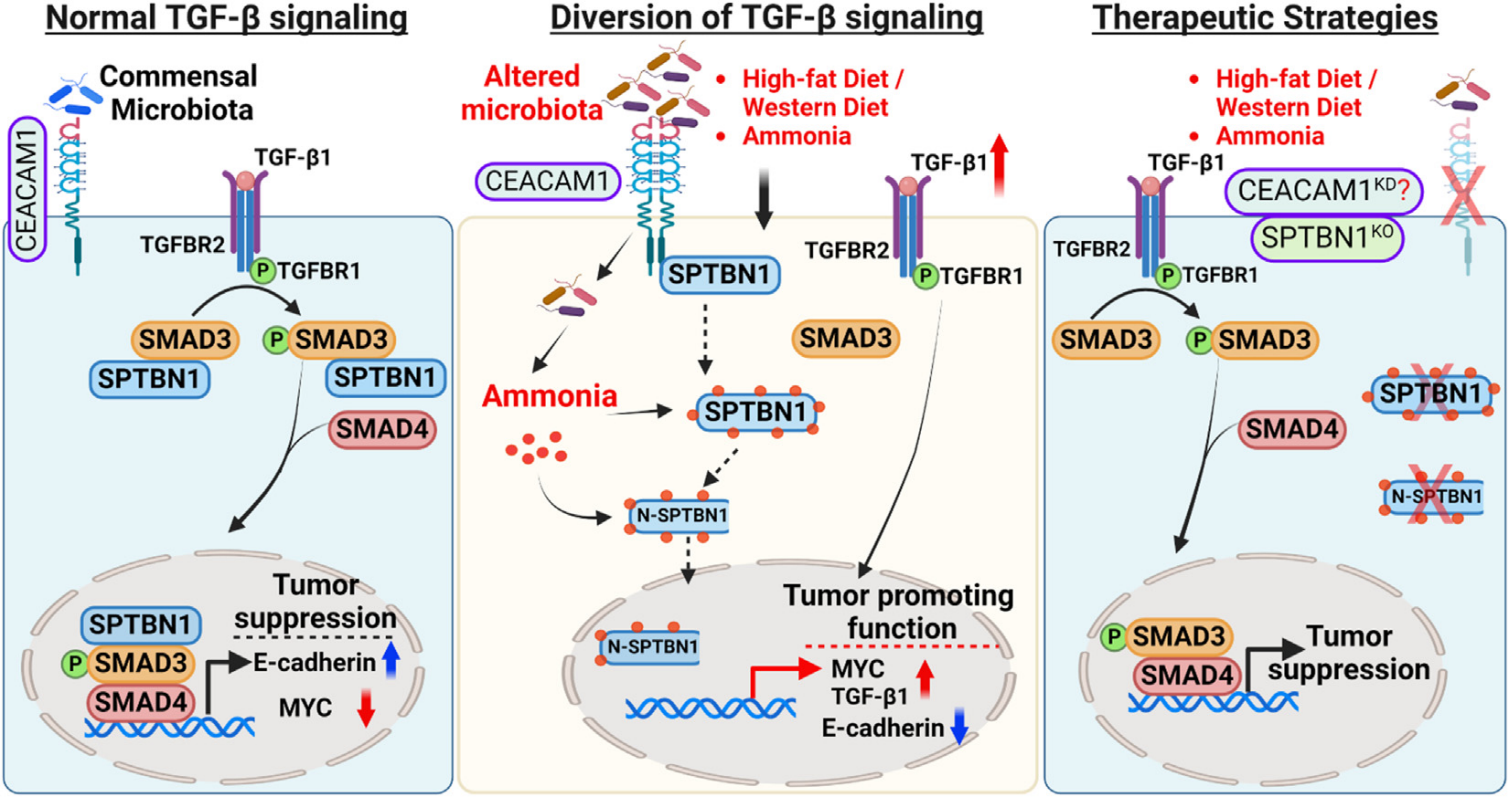
**Schematic representation of how ammonia modifies SPTBN1 cleaved products and disrupts normal SPTBN1–Smad3–TGF-β signaling to oncogenic signal.***Left*, normal regulation of the TGF-β pathway in intestinal epithelial cells, maintaining homeostasis. *Middle*, high-fat diet and other environmental toxins induces proinflammatory gut microbes, which utilize CEACAM1 for colonization, leading to an abnormal accumulation of toxic metabolites, such as ammonia. This ammonia interacts with cleaved fragments of the SMAD3/4 adaptor, SPTBN1, disrupting the normal SPTBN1–SMAD3 signaling and cause CRC. *Right*, our proposed therapeutic targets (SPTBN1 and CEACAM1), particularly targeting SPTBN1, can block these cleaved SPTBN1–ammonia adduct formation, thereby significantly inhibiting oncogenic signals and restoring tumor suppressor function of TGF-β signaling. CEACAM1, carcinoembryonic antigen–related cell adhesion molecule 1; CRC, colorectal cancer; SPTBN1, SMAD3 adaptor βII-spectrin; TGF-β, transforming growth factor beta.

In conclusion, this work indicates that disruption of the SMAD3 adaptor, βII-spectrin/TGF-β signaling pathway, exacerbated by a HFD or WD, significantly alters the gut microbiome, driving inflammation, and promoting oncogenesis. Further functional studies in the future could help us understand the in-depth intricacy of the loops between spectrin cleavage products and pathological interactions between CEACAM1 and gut microbes, leading to the production of toxic metabolites, such as ammonia, which impair tumor-suppressive TGF-β signaling and cause CRC (Fig. 6). These findings highlight βII-spectrin and CEACAM1 as promising molecular targets for therapeutic intervention in CRC, offering a potential strategy to restore normal signaling pathways and mitigate cancer progression in affected patients.

## Experimental procedures

### Data analysis

K–M curves of overall survival for the colon cancer patients (Sidra-LUMC AC-ICAM, n = 348) with or without Sptbn1 and SMAD3 genomic alterations were analyzed using cBioportal website tools (https://bit.ly/47EoUwA) (34). Co-occurrence of SMAD3 and SMAD4, and co-occurrence of CEACAM1 and SMAD4, was also analyzed using cBioportal website tools (https://bit.ly/3MWUQ5N). CEACAM1 protein expression values from the colon cancer patients and normal group (CPTAC data) were log2 normalized. *Z*-value for each sample was calculated as SDs from the median across samples. Correlations between CEACAM1 mRNA expression and overall survival in the colon cancers were analyzed using the K–M Plotter. Statistical significance was determined when *p* < 0.05.

### Microbiome DNA extraction, shotgun metagenomic sequencing, and bioinformatic analysis

Male and female C57BL/6 WT mice (n = 7) or obese TGF-β-driven mice (OTM) (*Aldh2*^−/−^*Sptbn1*^+/−^ mice) (n = 14) at 12 weeks old were fed with either HFD or control diet for 16 weeks (25). All animal experiments were conducted in accordance with National Institutes of Health guidelines and US federal laws and approved by the Institutional Animal Care and Use Committee at Feinstein Institutes for Medical Research, Northwell Health. DNA from fecal samples was extracted using the QIAmp PowerFecal DNA Kit (catalog no.: 12830-50; Qiagen). Concentration of dsDNA was determined using NanoDrop, and its quality was assessed using the Qubit dsDNA Broad Range DNA Assay Kit. Samples were prepared for Illumina sequencing according to the manufacturer's instructions using the Nextera XT DNA Library Preparation Kit (Illumina; FC-131-1096). Paired-end sequencing was carried out using a Mid Output v2.5 (300 cycles) kit (Illumina; 20024905) on a NextSeq 500, with dual indexing. Each dsDNA molecule was sequenced for 150 bases from both ends. Bioinformatics analysis was performed following protocol described elsewhere (17).

### Construction of expression plasmids

FL human SPTBN1 plasmid (pENTER-CMV-His-SPTBN1v1) was purchased from Charles River. N-SPTBN1 plasmid (1–4362 bp) was constructed using the following forward and reverse primer pair: 5′-GCTAGCGGACCGACGCGTAC-3′; reverse: 5′-TACCTCGTCGGTGCTCTTCC-3′. FL human CEACAM1 plasmid (pCMV3-SP-FLAG-CEACAM1) was purchased from Sino Biological (#HG10822-NF). CEACAM1-ΔC plasmid (1–1341 bp) was constructed using the following forward and reverse primer pair: 5′-TAATCTAGAGCGGCCGCCGA-3′; reverse: 5′-CAGAAAACATGCCAGGGCTA-3′. Reagents used for generating plasmids were purchased from NEB (#E0554S). All constructions and mutations were confirmed by sequencing.

### Cell culture, transfection, and siRNA-mediated silencing

Human CRC cell lines, SW480, Caco-2, and HCT116, were purchased from the American Type Culture Collection and cultured in Dulbecco's modified Eagle's medium/F12 medium supplemented with 10% fetal bovine serum and 1% streptomycin–penicillin in a 37 °C humidified atmosphere with 5% CO_2_.

For knockdown experiments, siRNA targeting *SPTBN1* (Dharmacon) was transfected into cells by Lipofectamine RNAiMAX (Thermo Fisher; catalog no.: 13778150) for 24 to 48 h (24). The sequences for si*SPTBN1* are si*SPTBN1*#1 5′-CCUGAAAGUGAGCGCAUUA-3′, si*SPTBN1*#2 5′-CCGCAUACGAGGAGCGUGU-3′, si*SPTBN1*#3 5′-GACAUGUCUUACGAUGAA-3′, and si*SPTBN1*#4 5′-GUGACAAGGCCGACGAUAU-3′.

For ammonia treatment, 2 × 10^4^ cells (HCT116/SW480) were plated in a 6-well plate, treated with vehicle control or NH_4_Cl (1, 5, or 10 mM) for 24 h. For TGF-β treatment, cells were treated with vehicle control or NH_4_Cl (10 mM) for 23 h, followed by treatment with TGF-β (200 pM) for 1 h.

### Total RNA sequencing

Total RNA was isolated from HCT116 cells treated with NH_4_Cl (10 mM) and TGF-β using Invitrogen PureLink RNA Mini Kit. Similarly, total RNA was extracted from siControl- or siSPTBN1-treated SW480 cells exposed to NH_4_Cl (10 mM) and TGF-β. Approximately 1 μg of RNA was used to create barcoded sequencing libraries, with library preparation conducted by Novogene Corporation. Genes that passed the quality check were quantified using HTSeq2 (Python package; v0.11.1). Differentially expressed mRNAs were identified using DESeq2 with the standard comparison mode between the four experimental conditions with multiple testing corrections. mRNA alterations were classified by hierarchical clustering. Heat map comparisons of three gene expression profiles from TGF-β, or NH_4_Cl (10 mM), or NH_4_Cl- and TGF-β-treated cells *versus* untreated control were generated.

### Immunoblotting and immunoprecipitation analyses

For Western blot analyses, cells were lysed with radioimmunoprecipitation assay lysis buffer (50 mM Tris–HCl, pH 7.4, 0.15 M NaCl, 0.25% deoxycholic acid, 1% NP-40, and 1 mM EDTA) containing cOmplete protease inhibitor and phosphatase inhibitor cocktail (Sigma–Aldrich; catalog no.: 4906845001 and catalog no.: 11836170001) followed by SDS-PAGE and immunoblotting using the following antibodies: anti-SPTBN1 (in-house, rabbit), anti-pSMAD3 (Cell Signaling; catalog no.: 9520, Research Resource Identifier [RRID]: AB_2193207), anti-SMAD3 (Cell Signaling; catalog no.: 9523, RRID: AB_2193182), anti-Caspase3 (Proteintech; catalog no.: 19677-1-AP, RRID: AB_10733244), anti-CEACAM1 (Santa Cruz; catalog no.: sc-166453, RRID: AB_2244678), and anti-GAPDH (Santa Cruz; catalog no.: sc-32233, RRID: AB_627679). After incubation with primary antibodies, the membrane was detected using horseradish peroxidase–conjugated secondary antibodies and a chemiluminescent substrate (catalog no.: 34577; SuperSignal West Pico PLUS Chemiluminescent Substrate, Thermo Fisher). Images were captured with the Azure Biosystem 300. The intensity of each band was measured using ImageJ software, and the data were analyzed with GraphPad Prism 9 (GraphPad Software, Inc).

For immunoprecipitation, cells were lysed with NP-40 lysis buffer (50 mM Tris–HCl, pH 7.5, 0.15 M NaCl, 1% NP-40, 1 mM EDTA) containing protease and phosphatase inhibitor cocktails. Whole cell lysate was precleared for 30 min to reduce nonspecific binding, followed by immunoprecipitation for overnight using Protein A/G Mix Magnetic Bead (MilliporeSigma; catalog no.: LSKMAGAG02) with anti-SPTBN1 (in house) or rabbit IgG (Cell Signaling; catalog no.: 2729) as control. Immunoprecipitated complexes (Magnetic Bead-lysate-antibody mix) were washed four times with NP-40 lysis buffer and eluted with sample buffer followed by immunoblotting analysis.

### Immunofluorescence analyses

SW480 colon cancer cells were seeded on coverslips placed in 6-well plates at a density of 1 × 10^5^ cells per well and incubated at 37 °C for 24 to 36 h. Once the cells adhered and reached appropriate confluency, they were transfected with siControl or si*SPTBN1* and incubated for 24 h. Post-transfection, cells were washed and cultured in serum-free Dulbecco's modified Eagle's medium (0% fetal bovine serum) for an additional 23 h with 10 mM NH_4_Cl treatment, followed by TGF-β (200 pM) stimulation for 1 h. Cells were fixed in 4% paraformaldehyde for 20 min, washed with PBS, and permeabilized with 0.5% Triton X-100 at room temperature for 20 min. Nonspecific binding was blocked by incubating the cells in 1% goat serum in PBS for 30 min. Immunofluorescent staining was performed using primary antibodies against SPTBN1 and SMAD3 (both at 1:100 dilution) overnight at 4 °C. The following day, cells were washed three times with PBS with Tween-20 and incubated with Alexa Fluor 488–conjugated anti-rabbit and Alexa Fluor 568–conjugated anti-mouse secondary antibodies, along with 4',6-diamidino-2-phenylindole for nuclear staining, for 1 h at room temperature. Confocal images were acquired using a 20X objective on a Zeiss LSM880 confocal microscope.

### AlphaFold 2 protein modeling and generation of SPTBN1–ammonia and SPTBN1–CEACAM1 complexes

Structural coordinates of SPTBN1 were sourced from the AlphaFold database (accession ID: Q01082) and subsequently refined using Swiss PDB Viewer to build the N-terminal domain spanning amino acids M1 to D1454 (47). For ammonia, three-dimensional structural coordinates were generated using ChemBioDraw Ultra 14.0 (48). Both the SPTBN1 and ammonia models underwent an energy minimization process before the docking calculations. InstaDock, v1.1, was used to facilitate the docking calculations for elucidating the interactions between ammonia and SPTBN1. The search space center was defined at coordinates X = −5.409, Y = 13.912, and Z = −5.417, with a spatial dimension of X = −5.409, Y = 13.912, and Z = −5.417, maintaining a 1 Å spacing during the blind docking process. This approach allowed ammonia to explore its potential binding sites on SPTBN1 freely. Subsequently, the initial docking results were screened to identify configurations exhibiting higher binding affinities, and the top-performing docked conformations were selected for in-depth interaction analysis. These chosen docked conformations were subjected to comprehensive interaction analysis using PyMOL to unravel their potential binding modes and interactions.

Molecular docking simulations were performed to unravel the possible structural mechanisms governing the interactions between SPTBN1 and the cytoplasmic domain of CEACAM1 (amino acid residues 453–526). A protein–protein docking approach was performed to investigate the binding pattern between CEACAM1_453–526 and SPTBN1. Structural coordinates of SPTBN1 were downloaded from the AlphaFold database (accession ID: Q01082), whereas the three-dimensional structural coordinates of CEACAM1_453–526 were modeled using c-QUARK. Subsequently, both structures underwent energy minimization in PyPAN using the GROMOS96 43B1 force field under vacuum conditions. HDOCK server was employed for the protein–protein docking simulations to assess the possible interactions between CEACAM1 and SPTBN1. Initial docking results were screened to identify those with the highest binding affinity, and the top-ranking docked conformations of CEACAM1_453–526 were selected for further analysis. PyMOL visualizer was employed to analyze the potential interactions within these selected conformations of CEACAM1 and SPTBN1.

## Data availability

RNA-sequencing datasets have been deposited to the Gene Expression Omnibus under Gene Expression Omnibus accession GSE281613.

## Supporting information

This article contains supporting information.

## Conflict of interest

The authors declare that they have no conflicts of interest with the contents of this article.
