# Supplementary material for: Microbial metabolite ammonia disrupts TGF-β signaling to promote colon cancer
Source: J Biol Chem. 2025 Apr 29;301(6):108559. doi: 10.1016/j.jbc.2025.108559 (PMC12155590; doi:10.1016/j.jbc.2025.108559)
Supplement: Supporting Information [file mmc1.docx]

**Microbial metabolite ammonia disrupts TGF-β signaling to promote colon cancer**

Krishanu Bhowmick^1,2*^, Xiyan Xiang^1,2^, Xiaochun Yang^1,2^, Taj Mohammad^3^, Christine L. Molmenti^4,5,6^, Bibhuti Mishra^1,7^, Srinivasan Dasarathy^8^, Adrian R. Krainer^2^, Md. Imtaiyaz Hassan^3^, Keith A. Crandall^9^, Lopa Mishra^1,2,10*^

^1^Institute for Bioelectronic Medicine, Feinstein Institutes for Medical Research; Divisions of Gastroenterology and Hepatology, Department of Medicine, Northwell Health, Manhasset, NY, USA.

^2^Cold Spring Harbor Laboratory; Cold Spring Harbor, NY, USA.

^3^Centre for Interdisciplinary Research in Basic Sciences, Jamia Millia Islamia, Jamia Nagar, New Delhi, India.

^4^ Department of Occupational Medicine, Epidemiology and Prevention, Zucker School of Medicine at Hofstra/Northwell, Hempstead, NY, USA

^5^ Feinstein Institutes for Medical Research, Institute of Cancer Research, Manhasset, NY, USA ^6^Department of Surgery, Northwell Health, Manhasset, NY, USA

^7^Donald and Barbara Zucker School of Medicine at Hofstra/Northwell Health, Department of Neurology, Hempstead, NY, USA

^8^Division of Gastroenterology and Hepatology, Cleveland Clinic, Cleveland, OH, USA

^9^Computational Biology Institute and Department of Biostatistics and Bioinformatics, Milken Institute School of Public Health, George Washington University, Washington DC, USA

^10^Department of Surgery, George Washington University, Washington DC, USA.

*Corresponding Author

***** To whom correspondence should be addressed: [lopamishra2@gmail.com](mailto:lopamishra2@gmail.com) and [bhowmick.krishanu@gmail.com](mailto:bhowmick.krishanu@gmail.com)

Supplementary Figures S1-S7

**
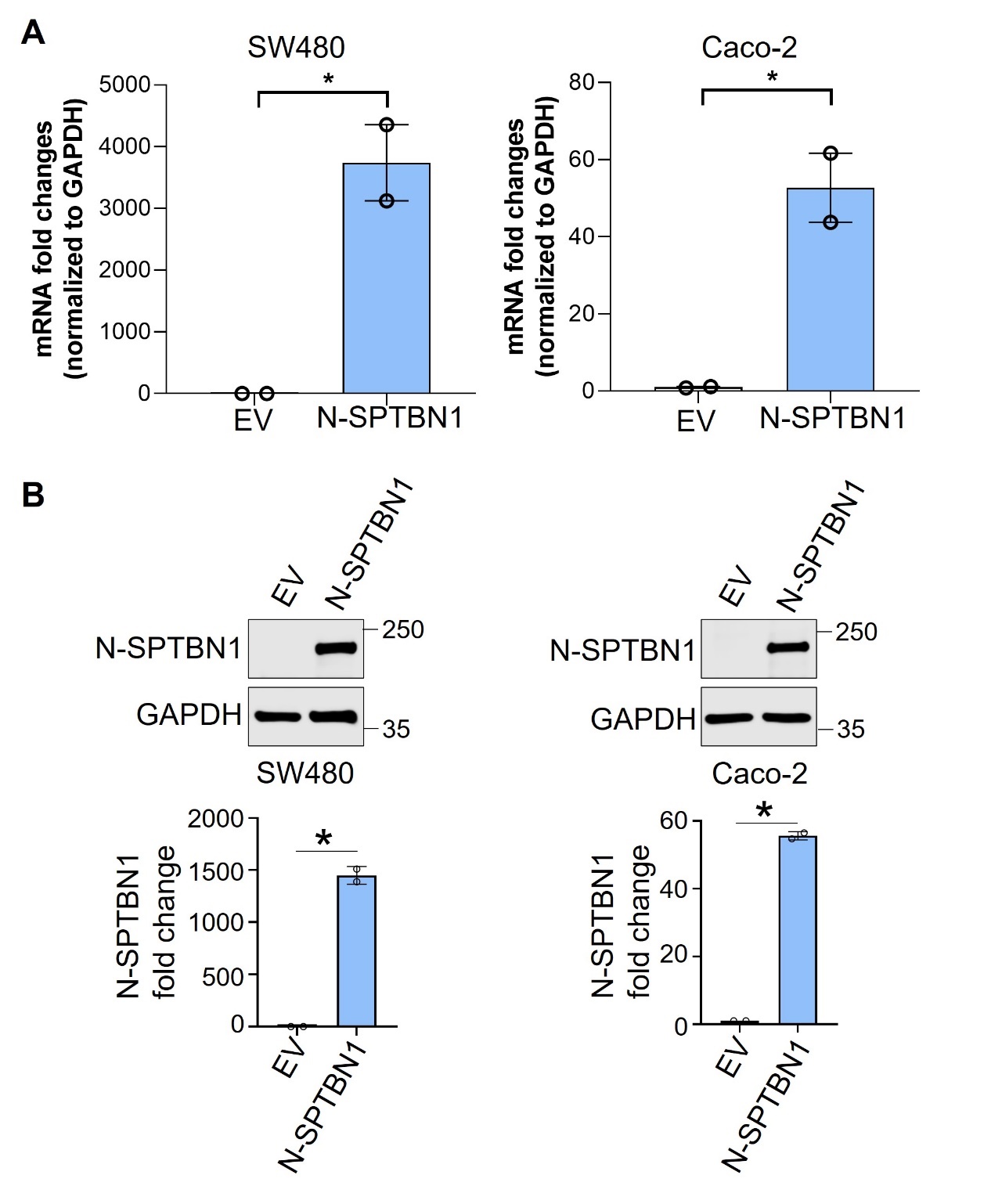
**

**Supplementary Figure 1.** Transfection efficiency of N-SPTBN1 in SW480 and Caco-2 human colon cancer cells. SW480 and Caco-2 cells were transfected with either empty vector (EV) or an N-SPTBN1 expression construct. Cells were harvested 48 hours post-transfection to assess transfection efficiency. (A) Quantitative RT-PCR analyses showing that N-SPTBN1 mRNA levels are significantly elevated in transfected cells compared to EV controls. (B) Western blot analyses demonstrating robust N-SPTBN1 protein expression in both cell lines. Data are representative of two independent experiments. Statistical significance was determined using an unpaired two-tailed Student’s t-test (**P < 0.05*).


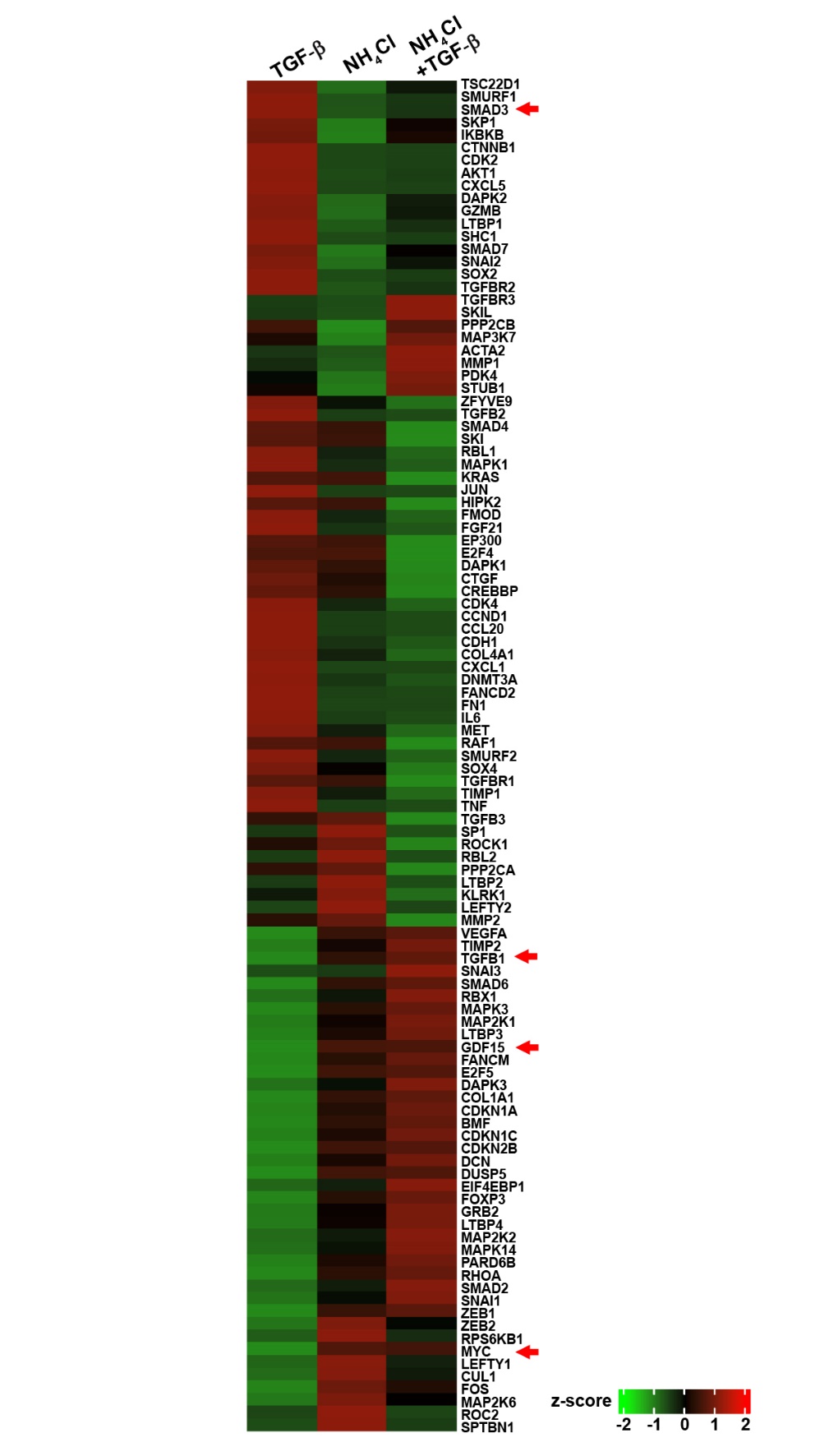


**Supplementary Figure 2. Ammonia-disrupts TGF-β signaling.** RNA sequencing analyses reveal dysregulation in TGF-β signaling upon ammonia treatment. Heatmap shows log2 fold changes in expressions of genes involved in the TGF-β signaling in HCT116 human CRC cells treated with TGF-β, NH_4_Cl, and NH_4_Cl-TGF-β compared to untreated cells.


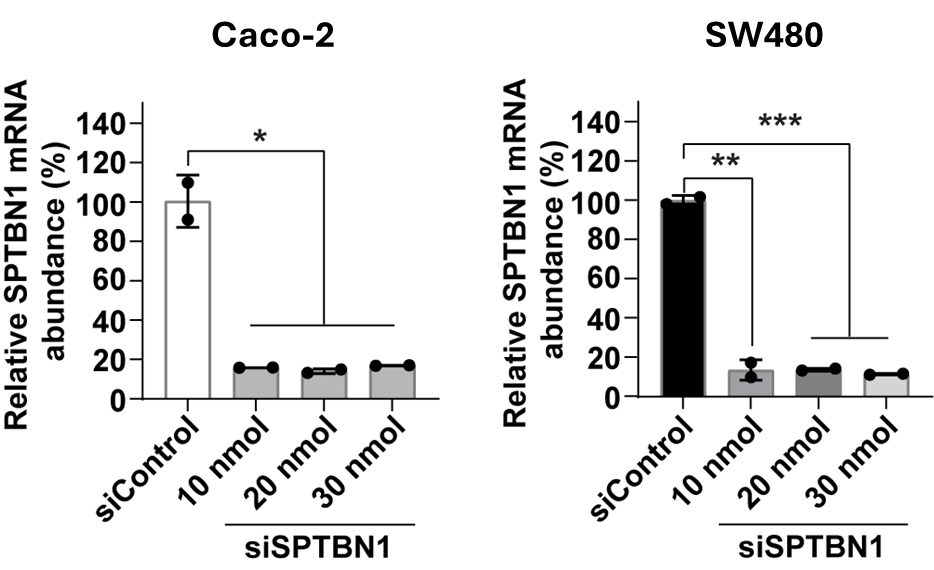


**Supplementary Figure 3.** siSPTBN1 efficiently reduces SPTBN1 expression in Caco-2 and SW480 cells. qRT-PCR analysis shows a significant reduction in SPTBN1 mRNA levels in siSPTBN1-transfected Caco-2 (left panel) and SW480 (right panel) human colon cancer cells compared to siControl. Expression is normalized to GAPDH. Error bars: ± SD; **p < 0.05*, ***p < 0.005*, ****p < 0.001*.


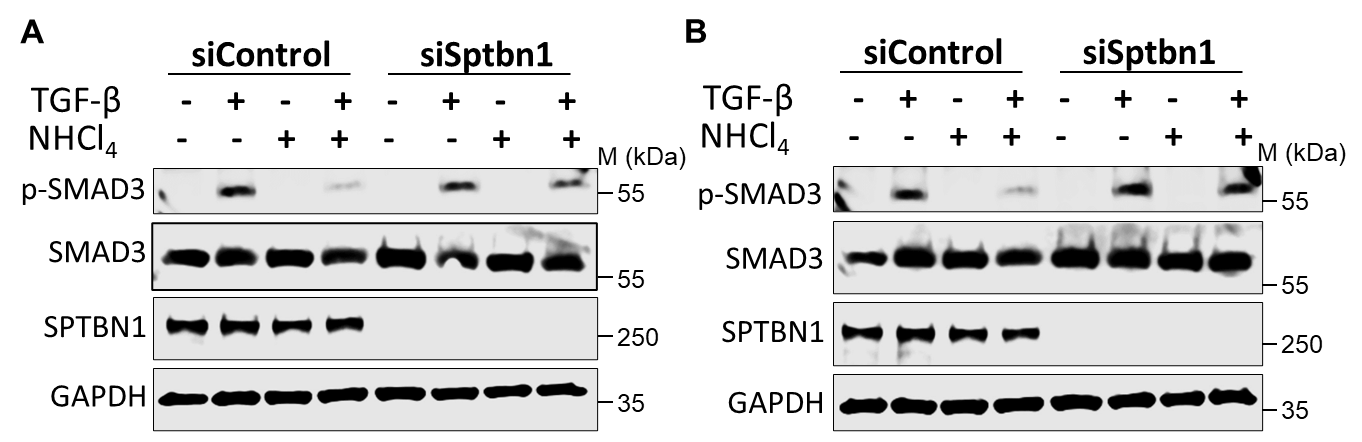


**Supplementary Figure 4.** SPTBN1 knockdown using siRNA in human SW480 colon cancer cells blocking ammonia toxicity and restores TGF-β signaling in human SW480 CRC cells.


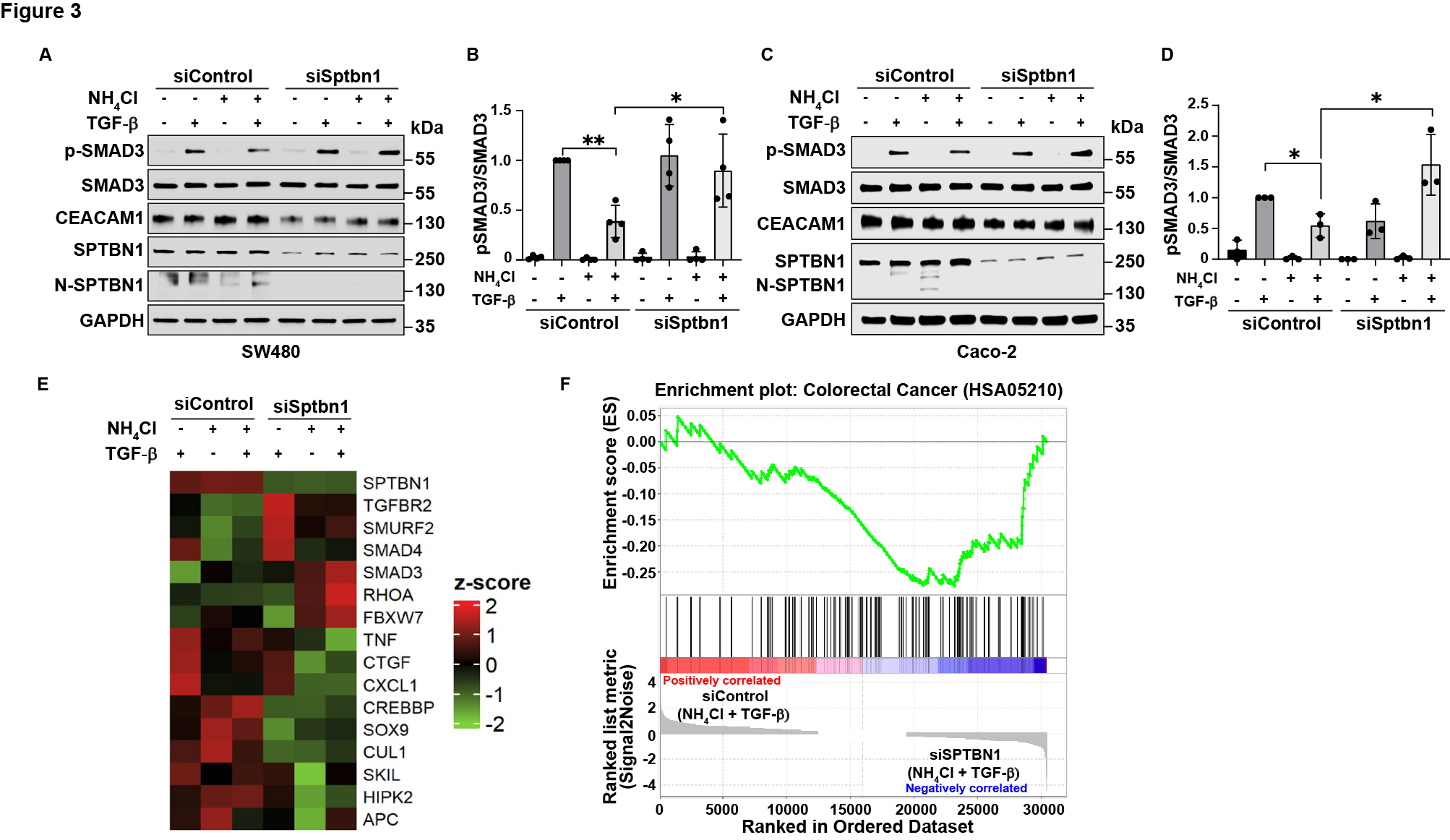


**Supplementary Figure 5.** SPTBN1 silencing using siRNA inhibits the transcription of colorectal cancer inducing genes. GSEA analyses of the KEGG colorectal cancer pathway (HSA05210) showing negative enrichment in siSPTBN1-treated cells compared to siControl under NH_4_Cl and TGF-β, suggesting that SPTBN1 knockdown attenuates oncogenic programs by restoring tumor-suppressive TGF-β/SMAD3 signaling.


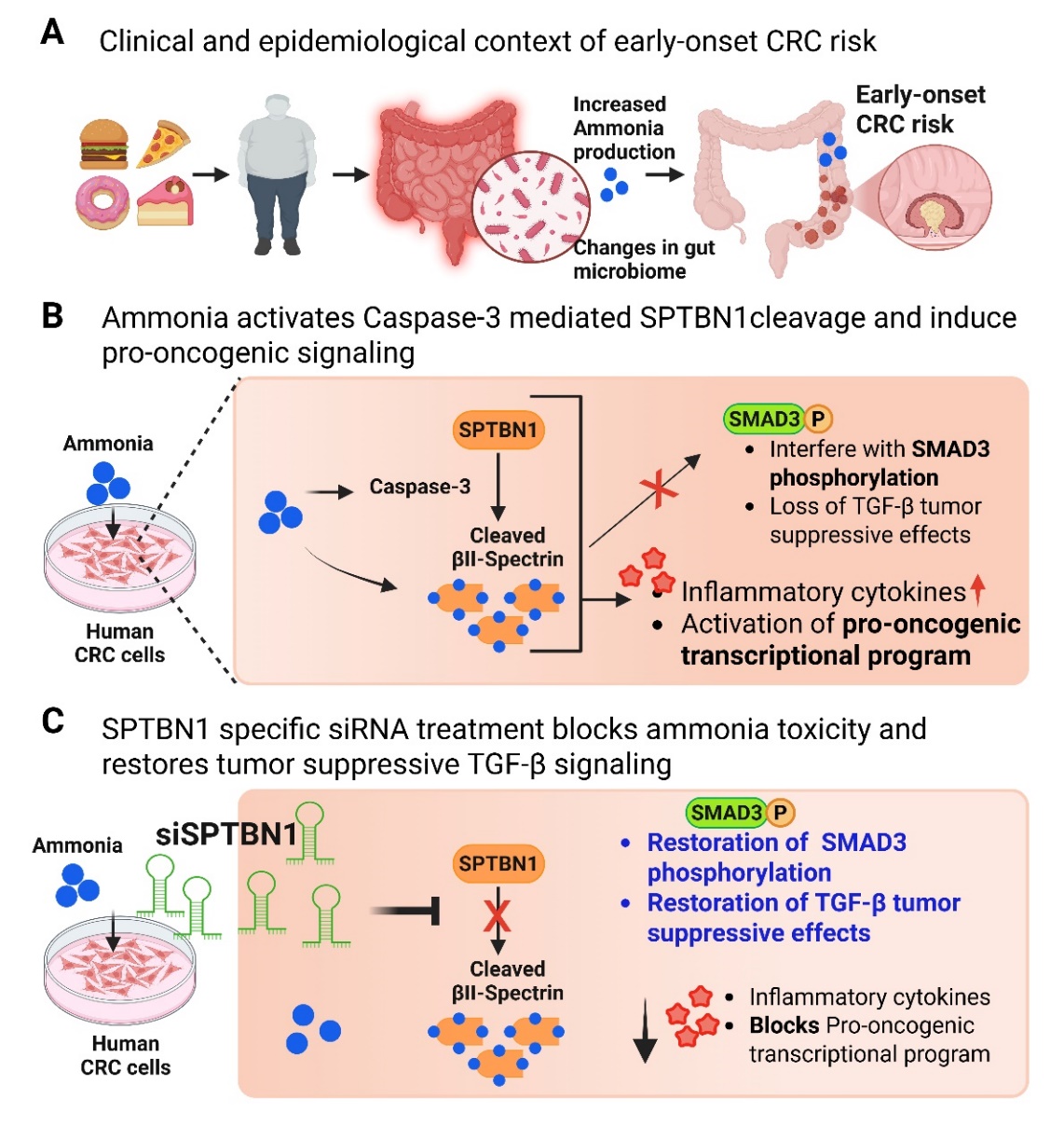


**Supplementary Figure 6. Diagram depicting how microbial metabolite ammonia cleaves SPTBN1, disrupts TGF-β signaling and promotes pro-oncogenic signaling in colorectal cancer.**

(A) Overview of the clinical and epidemiological context of early-onset colorectal cancer (CRC) development. Western diets/High-fat diets promote obesity and gut microbial changes in obese individuals, leading to the accumulation of microbial metabolite ammonia in the gut. Raised ammonia levels in the intestinal lumen are associated with an increased risk of early-onset CRC.

(B) Ammonia exposure in human CRC cells activates caspase-3, leading to cleavage of the SMAD3 adaptor protein SPTBN1 (βII-spectrin). Cleaved SPTBN1 fragments interfere with SMAD3 phosphorylation, disrupting tumor-suppressive TGF-β signaling, releasing inflammatory cytokines, and activating the pro-oncogenic transcriptional program.

(C) siRNA-mediated knockdown of SPTBN1 (siSPTBN1) in human CRC cells blocks ammonia-induced accumulation of SPTBN1 cleaved fragments and restores SMAD3 phosphorylation and tumor suppressive TGF-β signaling.


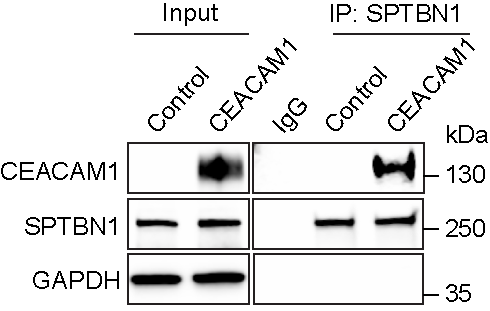


**Supplementary Figure 7.** Co-immunoprecipitation assay demonstrating the interaction between SPTBN1 and CEACAM1. HCT116 cells were transfected with a full-length CEACAM1 and SPTBN1 plasmids, and cell lysates collected after 24 hours were immunoprecipitated with SPTBN1 antibodies, followed by immunoblotting with the indicated antibodies.
